# Long‐term efficacy and safety of bexarotene for Japanese patients with cutaneous T‐cell lymphoma: The results of a phase 2 study (B‐1201)

**DOI:** 10.1111/1346-8138.14923

**Published:** 2019-05-15

**Authors:** Toshihisa Hamada, Yoshiki Tokura, Makoto Sugaya, Mikio Ohtsuka, Ryoji Tsuboi, Tetsuo Nagatani, Eiji Kiyohara, Mamori Tani, Mitsuru Setoyama, Shigeto Matsushita, Kazuhiro Kawai, Kentaro Yonekura, Toshiaki Saida, Keiji Iwatsuki

**Affiliations:** ^1^ Department of Dermatology Okayama University Graduate School of Medicine, Dentistry and Pharmaceutical Sciences Okayama Japan; ^2^ Department of Dermatology Takamatsu Red Cross Hospital Takamatsu Japan; ^3^ Department of Dermatology Hamamatsu University School of Medicine Shizuoka Japan; ^4^ Department of Dermatology International University of Health and Welfare Chiba Japan; ^5^ Department of Dermatology Fukushima Medical University Fukushima Japan; ^6^ Department of Dermatology Tokyo Medical University Tokyo Japan; ^7^ Department of Dermatology Tokyo Medical University Hachioji Medical Center Tokyo Japan; ^8^ Department of Dermatology Graduate School of Medicine Osaka University Osaka Japan; ^9^ Department of Dermatology Faculty of Medicine University of Miyazaki Miyazaki Japan; ^10^ Department of Dermatology Kagoshima University Graduate School of Medical and Dental Sciences Kagoshima Japan; ^11^ Department of Dermato‐Oncology/Dermatology National Hospital Organization Kagoshima Medical Center Kagoshima Japan; ^12^ Department of Dermatology Imamura General Hospital Kagoshima Japan; ^13^ Department of Dermatology Shinshu University Nagano Japan

**Keywords:** adverse event, bexarotene, cutaneous T‐cell lymphoma, mycosis fungoides, objective response rate

## Abstract

The present study (B‐1201 clinical trial) was conducted as a multicenter, open‐label, single‐arm phase II study to evaluate the long‐term safety, tolerability and efficacy of bexarotene. This study enrolled 10 Japanese adults aged more than 20 years with cutaneous T‐cell lymphoma (CTCL) who completed the 24‐week study period of the B‐1101 trial. The objective response rate (ORR) was 53.8% (95% confidence interval, 25.1–80.8). In the early stage (IB), the ORR was 60% (3/5 cases). In the advanced stage (IIB and IIIA), the ORR was 57.1% (4/7 cases). The median time to response was 58 days (range, 27–168). The median treatment duration was 380 days (range, 33–1674). The median duration of response (DOR) could not be reached during the study period. The longest DOR reached 1618 days at the end of the B‐1201 trial. Nine patients (56.3%) in the full analysis set (FAS) population experienced dose reduction of bexarotene. Common drug‐related adverse events in the FAS population included hypothyroidism (93.8%), hypertriglyceridemia (81.3%), hypercholesterolemia (81.3%), leukopenia (68.8%) and neutropenia (56.3%). Dose‐limiting toxicity (DLT) was present in five (38.5%) of the 13 patients in the 300 mg/m^2^ cohort. Of the five patients, four developed grade 3 neutropenia and one developed grade 4 hypertriglyceridemia. All DLT cases recovered after the discontinuation of bexarotene. None of the five patients discontinued this trial because of DLT. The B‐1201 trial shows the long‐term safety of oral bexarotene for Japanese patients with CTCL, despite frequent dose reduction.

## Introduction

Cutaneous lymphomas (CL) are a heterogeneous group of extranodal non‐Hodgkin's lymphomas, defined as lymphomas with skin infiltration of neoplastic lymphocytic cells without nodal or internal involvement at diagnosis.^1^ Mycosis fungoides (MF) is the most common type of CL throughout the world as well as in Japan.^1^, ^2^ Based on the established version of clinical staging in MF/Sézary syndrome (SS),^3^, ^4^ patients with advanced stage MF/SS (IIB–IVB) have shown a poorer prognosis than those with early stage MF/SS (IA–IIA).^5^, ^6^ Clinical guidelines for cutaneous T‐cell lymphoma (CTCL) including MF/SS are now available, and they show the current therapeutic options.^7^, ^8^, ^9^ However, many different recommendations for the treatment of CTCL have been launched in various areas or nations of the world, especially for advanced‐stage disease.^10^

In Japan, skin‐direct therapies such as topical corticosteroids and photo‐(chemo)therapy is recommended as an initial treatment for CTCL patients.^8^, ^11^ Patients with advanced‐stage CTCL may require various combinations, mainly including radiation therapy, biological response modifiers and chemotherapy. Recent advances in elucidating biophysiological characteristics or novel molecular targets on CTCL have promoted new therapeutic modalities such as histone deacetylase inhibitors, molecular‐targeted agents and purine nucleoside phosphorylase inhibitors.^12^, ^13^, ^14^, ^15^

Bexarotene, a synthesized retinoid X receptor (RXR) agonist, is a widely used drug for patients with CTCL, and is available in Japan. We have already reported the efficacy, safety and tolerability of bexarotene for Japanese patients with CTCL as a result of a multicenter, open‐label, single‐arm phase I/II clinical study (B‐1101 trial).^16^ In the B‐1101 trial, a favorable response and common drug‐related adverse events (AE) including hypothyroidism (92%), hypercholesterolemia (77%) and hypertriglyceridemia (77%) were demonstrated at 24 weeks of the clinical trial.

We conducted a multicenter, open‐label, single‐arm phase II clinical trial (B‐1201 trial) as a long‐term study after the B‐1101 trial in order to investigate the long‐term efficacy and safety of bexarotene for Japanese patients with CTCL.

## Methods

### Study design

**Figure 1:**
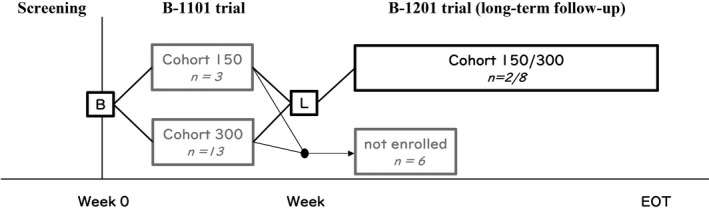
Study flowchart. The B‐1201 trial was planned as a long‐term follow‐up study of the B‐1101 trial. Ten patients who completed the 24‐week study period of the B‐1101 trial and met the eligibility criteria enrolled in this study. In the B‐1201 trial, two of the 10 patients originated from the 150 mg/m^2^ cohort of the B‐1101 trial. The residual eight originated from the 300 mg/m^2^ cohort. B, baseline; EOT, end of treatment; L, starting point of long‐term trial.

The B‐1201 trial was a multicenter, open‐label, single‐arm phase II study to assess the tolerability, safety and efficacy of long‐term bexarotene therapy between 1 August 2012 and 10 January 2017. The present trial was planned as a long‐term follow‐up study after the previous B‐1101 trial.^16^ At the end of the week 24 visit in the B‐1101 trial, patients were eligible to continue bexarotene for up to 4 additional years (Fig. 1).

All patients provided informed consent, and the study was conducted according to Good Clinical Practice guidelines and the Declaration of Helsinki. This study was registered in the University Hospital Medical Information Network (no. 000006744).

At the beginning of this study, oral bexarotene was maintained at the same dose as used in the B‐1101 trial, and the dose was changed if necessary.

### Patients

This clinical study enrolled Japanese adults aged 20 years or older with CTCL who had completed the 24‐week study period in the B‐1101 trial. Patients who met the eligibility criteria and agreed to participate in writing were included in this study. The key exclusion criteria included progressive disease (PD) as determined using the modified Severity‐Weighted Assessment Tool (mSWAT) at the end of the week 24 visit in the B‐1101 trial.

The full analysis set (FAS) includes all CTCL patients who received at least one dose of the B‐1101 trial medication (i.e. the 16 subjects shown in Table 1).

**Table 1:** List of 16 Japanese patients (FAS population) in B‐1101 and B‐1201 trials

Trial	Patient/sex/age	Cohort	CTCL type	Stage	mSWAT (baseline)	Tx duration (day)	Final dose (mg/m^2^)	TTR (day)	Response
B‐1101	B04‐01/M/32	300	ALCL	–	0.2	29	300	–	PD
	B09‐03/M/74	300	MF	IIB	49.1	114	300	–	SD
	B01‐01/M/55	300	MF	IB	43.2	161	100	–	SD
	B02‐01/M/61	150	MF	IB	37.8	167	150	139	PR
	B09‐02/M/61	300	MF	IB	34.0	170	200	86	PD
	B08‐01/F/47	300	MF	IB	12.4	170	300	56	PR
B‐1201	B05‐01/M/64	300	MF	IB	41.9	285	300	28	PR
	B02‐02/M/74	300	MF	IIB	18.9	347	300	54	PR
	B07‐01/F/38	150	MF	IIA	17.2	413	150	–	PD
	B05‐02/F/53	300	MF	IIB	20.2	426	200	27	PR→SD†
	B10‐01/M/59	300	MF	IIB	32.5	659	100	113	PR
	B07‐04/F/34	300	MF	IB	15.2	673	100	28	PR
	B03‐01/F/65	300	MF	IIIA	99.0	764	200	168	PR→SD†
	B07‐02/M/52	150	MF	IB	9.3	1338	100	56	PR→CR‡
	B07‐03/F/59	300	MF	IIIA	72.3	1359	100	58	PR
	B06‐01/M/47	300	MF	IIIA	95.0	1674	200	56	PR

^†^Two patients who had achieved PR in the B‐1101 trial resulted in SD during the B‐1201 study period. ^‡^One patient in the 150 mg/m^2^ cohort achieved CR at week 40 of the B‐1201 trial. ALCL, primary cutaneous anaplastic large cell lymphoma; CR, complete response; CTCL, cutaneous T‐cell lymphoma; FAS, full analysis set; MF, mycosis fungoides; PD, progressive disease; PR, partial response; SD, stable disease; TTR, time to response; Tx, treatment.

### Efficacy analysis

The key efficacy end‐point was the evaluation of skin responses using mSWAT as previously described.^3^, ^4^, ^16^ The objective response rate (ORR) was defined as the percentage of patients with partial response (PR) and complete response (CR), in the same manner as the B‐1101 trial.^16^ The 95% confidence interval (CI) of the ORR was calculated.

Other efficacy end‐points included the Physician Global Assessment (PGA), composite assessment of index lesion severity (CA), primary end‐point classification (PEC), time to response (TTR), duration of response (DOR) and time to disease progression (TTP). The evaluation of bexarotene efficacy using PGA, CA and PEC has been reported in previous clinical studies.^17^, ^18^ The above‐mentioned efficacy end‐points were analyzed among the FAS population.

### Safety analysis

The baseline status of the B‐1101 trial was applied to the present trial on the following items: vital signs, patient history, Eastern Cooperative Oncology Group performance status, digital photography, hematology, blood chemistry, urinalysis, 12‐lead electrocardiogram, chest radiographs, computed tomography of the abdomen and pelvis, and ophthalmologic slit lamp examination.

Safety assessments involved the collection of data on AE, clinical laboratory tests, physical examination and vital signs in the same manner as in the B‐1101 trial, and were analyzed among the FAS population.

The severity grade (Common Terminology Criteria for Adverse Events version 4.0 – Japan Clinical Oncology Group) and the relationship to bexarotene were determined for each AE.

### Statistical analysis

The key secondary end‐point was assessed in all patients who achieved an objective response (OR). TTR was defined as the time from the initiation of treatment to the first confirmed OR. DOR was defined as the time between the first response and PD. TTP was the interval from the first day of the treatment to the first observation when the patient met the criteria for PD. The Kaplan–Meier method was used to estimate the median of TTR, DOR and TTP.

## Results

### Patient characteristics

Overall, 10 patients in the B‐1101 trial met the key criteria and entered the B‐1201 trial. The characteristics of the FAS population are shown in Table 1. The 16 CTCL patients included one with primary cutaneous anaplastic large cell lymphoma and 15 with MF. The mean baseline mSWAT score was 37.4, ranging 0.2–99.0 (Table 1).

The B‐1101 trial included two cohorts as follows: (i) three patients received oral bexarotene 150 mg/m^2^; and (ii) 13 received 300 mg/m^2^. In the B‐1201 trial, two of the 10 originated from the 150 mg/m^2^ cohort and the residual eight from the 300 mg/m^2^ cohort. In the B‐1201 trial, all 10 patients enrolled had MF (Table 1).

### Efficacy

The ORR in the FAS population was 56.3% (95% CI, 29.9–80.2). The ORR of the 300 mg/m^2^ cohort was 53.8% (95% CI, 25.1–80.8). In the five MF patients with the early stage (IB), the ORR was 60%. In the seven MF patients with advanced stage (IIB and IIIA), the ORR was 57.1%.

In the B‐1201 trial, six of the eight patients in the 300 mg/m^2^ cohort and one of the two patients in the 150 mg/m^2^ cohort achieved OR, respectively (Table 1). Two patients who had achieved PR in the B‐1101 trial failed to meet the OR criteria after the initiation of the B‐1201 trial (Table 1). Only one patient in the 150 mg/m^2^ cohort achieved CR at week 40 of the B‐1201 trial (Table 1).

**Figure 2:**
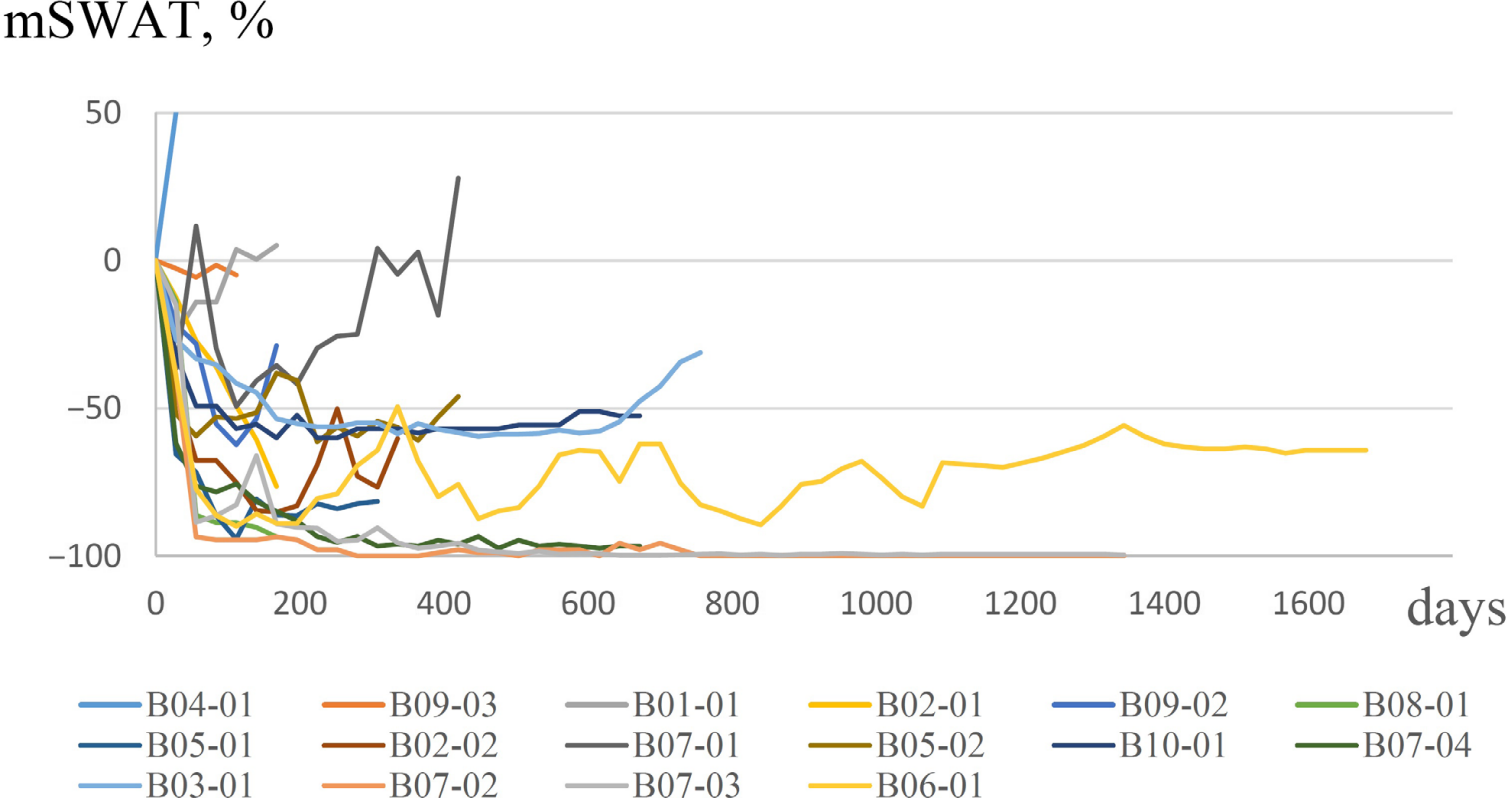
Spider plot of mSWAT. Spider plot of skin disease changes during the trial using mSWAT. Longitudinal responses are shown in reference of baseline (% mSWAT as 0). mSWAT, the modified Severity‐Weighted Assessment Tool.

The median TTR was 58 days, ranging 27–168 days (Table 1). The median treatment duration was 380 days, ranging 33–1674 days (Table 1). Of note, three patients had maintained the treatment for over 1300 days (Fig. 2). The median DOR could not be reached during the study period. The longest DOR reached 1618 days at the end of the B‐1201 trial.

### Safety

Adverse events related to bexarotene are shown in Table 2. Hypothyroidism (12/13, 92.3%), hypertriglyceridemia (11/13, 84.6%) and hypercholesterolemia (10/13, 76.9%) occurred in the 300 mg/m^2^ cohort. Also, these drug‐related AE were frequently observed in the 150 mg/m^2^ cohort (hyperthyroidism [3/3, 100%], hypercholesterolemia [3/3, 100%], hypertriglyceridemia [2/3, 67%]).

**Table 2:** Treatment‐related adverse events (AE) occurring in more than 10% of patients

Cohort	150 mg†	300 mg			FAS population		
Trial no.	B‐1101	B‐1101	B‐1201	Total	*n* (%)	Time to AE (median, days)	Duration of AE (median, days)
	*n* (%)	*n* (%)	*n* (%)	*n* (%)			
All AE	3 (100)	13 (100)		13 (100)	16 (100)		
Hypothyroidism	3 (100)	12 (92.3)		12 (92.3)	15 (93.8)	8	218
Hypercholesterolemia	3 (100)	10 (76.9)		10 (76.9)	13 (81.3)	8	127
Hypertriglyceridemia	2 (66.7)	10 (76.9)	1 (7.7)	11 (84.6)	13 (81.3)	8	50
White blood cells decreased	1 (33.3)	9 (69.2)	1 (7.7)	10 (76.9)	11 (68.8)	13	37
Neutrophil count decreased		8 (61.5)	1 (7.7)	9 (69.2)	9 (56.3)	15	15
AST increased	2 (66.7)	2 (15.4)		2 (15.4)	4 (25.0)	8	18
ALT increased	2 (66.7)	1 (7.7)		1 (7.7)	3 (18.8)	8	15
Platelet count increased	1 (33.3)	2 (15.4)		2 (15.4)	3 (18.8)	15	178
Anemia		3 (23.1)		3 (23.1)	3 (18.8)	43	228
Headache		2 (15.4)		2 (15.4)	2 (12.5)	1	30.5
Nausea		2 (15.4)		2 (15.4)	2 (12.5)	33	10.5
Vomiting		2 (15.4)		2 (15.4)	2 (12.5)	33	4.5
Alopecia		1 (7.7)	1 (7.7)	2 (15.4)	2 (12.5)	719	360
Renal dysfunction		1 (7.7)	1 (7.7)	2 (15.4)	2 (12.5)	101.5	151.5
Malaise		2 (15.4)		2 (15.4)	2 (12.5)	5.5	269.5
Hyperuricemia	1 (33.3)				1 (6.3)	15	87
Sinus arrhythmia	1 (33.3)				1 (6.3)	169	169
APTT extension	1 (33.3)				1 (6.3)	15	750
QT prolongation	1 (33.3)				1 (6.3)	169	269
ALP increased	1 (33.3)				1 (6.3)	77	148

^†^No patient in the 150 mg/m^2^ cohort developed treatment‐related AE during the B‐1201 study period. ALT, alanine aminotransferase; APTT, activated partial thromboplastin time; AST, aspartate aminotransferase; FAS, full analysis set.

Leukopenia (10/13, 76.9%) and neutropenia (9/13, 69.2%) frequently occurred in the 300 mg/m^2^ cohort. Both AE infrequently occurred in the 150 mg/m^2^ cohort (Table 2). The median time to AE including hypothyroidism, hypertriglyceridemia and hypercholesterolemia was 8 days. The median time to AE of neutropenia and leukopenia was 13 and 15 days, respectively. The median duration of these AE ranged 15–218 days (Table 2).

The AE observed during the B‐1201 study period were almost the same as those in the B‐1101 trial. The newly emerging drug‐related AE in the B‐1201 trial included neutropenia, stomatitis, impaired renal function, hair loss, cataract, dizziness, skin thinning, sinus arrhythmia, activated partial thromboplastin time shortening and electrocardiogram QT prolongation.

Dose‐limiting toxicity (DLT) was present in five out of the 13 patients (38.5%) in the 300 mg/m^2^ cohort (Table 3). No DLT was observed in the 150 mg/m^2^ cohort. During the B‐1201 study period, four of the eight patients developed grade 3 neutropenia and one developed grade 4 hypertriglyceridemia as DLT. All DLT cases recovered as a result of the discontinuation of bexarotene. No patient discontinued this trial because of DLT.

**Table 3:** Drug‐related grade 3 or 4 adverse events (AE)

Patient	AE	Time to AE (day)	Grade	DLT y/n	Dose (mg/m^2^)	Recover y/n	Duration of AE (day)
B01‐01	Dyslipidemia	8	3	n	300	y	106
	Neutrophil count decreased	15	3	y	300	y	29
B03‐01	Neutrophil count decreased†	414	3	y	300	y	50
B05‐02	Hypertriglyceridemia	7	3	n	300	y	29
B06‐01	Hypercholesterolemia	6	3	n	300	n	1696
	ALT increased	8	3	y	300	y	21
	AST increased	8	3	y	300	y	21
B07‐02	Hypertriglyceridemia	8	3	n	150	y	374
B07‐03	Neutrophil count decreased	15	3	y	300	y	45
B07‐04	Hypertriglyceridemia	8	3	n	200	y	50
B09‐02	Hypertriglyceridemia	8	3	n	300	y	183
B10‐01	Hypertriglyceridemia	4	4	y	300	y	26
	Neutrophil count decreased†	560	3	y	100	y	33

^†^AE newly experienced during the B‐1201 study period. DLT, dose‐limiting toxicity; n, no; y, yes.

Drug‐related grade 3 or 4 AE developed in nine patients in the FAS population (Table 3). In two of the nine patients, grade 3 neutropenia newly occurred as DLT on days 414 and 560 in the B‐1201 trial, respectively (Table 3).

Severe AE were found in three patients in the 300 mg/m^2^ cohort, and included bile duct stones (one case), excessive drug intake (one case) and hypertriglyceridemia (three cases). No patients died during the study periods of the B‐1101 and B‐1201 trials.

### Dose of bexarotene

The final dose of bexarotene is summarized in Table 1. Nine patients (56.3%) in the FAS population experienced a dose reduction of bexarotene. The nine patients included eight of the 13 patients (61.5%) in the 300 mg/m^2^ cohort and one of the three patients (33.3%) in the 150 mg/m^2^ cohort. Of the 10 patients in the B‐1201 trial, only two of the eight patients in the 300 mg/m^2^ cohort and one of the two in the 150 mg/m^2^ cohort had maintained the initial dose of bexarotene. Eventually, in four of the 10, the bexarotene dose was reduced to 100 mg/m^2^ (Table 1).

## Discussion

In the B‐1101 trial, the ORR of the 300 mg/m^2^ cohort was 61.5% by mSWAT in the 24‐week therapeutic period.^16^ In the present long‐term follow‐up study (the B‐1201 trial), the ORR of the 300 mg/m^2^ cohort was reduced to 53.8%, because two patients who had achieved PR in the B‐1101 trial could not meet the OR criteria during the B‐1201 study period.

The longest DOR reached 1618 days from the initiation of the B‐1101 trial. Dose reduction of bexarotene was frequently observed in nine (56.3%) patients in the FAS population, particularly in eight (61.5%) of the 13 patients in the 300 mg/m^2^ cohort.

The median treatment duration reached over a year, even in the FAS population. These results suggest that bexarotene may show a long‐term efficacy for CTCL patients despite frequent dose reduction. The results of the B‐1201 trial revealed that three patients had maintained the treatment for over 1300 days. The DOR can last years in Japanese responders as shown in the previous results.^18^, ^19^

The median TTR was 58 days, which was close to that of the previous clinical trial for the early stage CTCL at 57 days.^17^ When bexarotene is introduced, a 2‐month follow up is recommended to validate its efficacy.

### Hypothyroidism

Central hypothyroidism frequently occurs in CTCL patients treated with bexarotene.^16^, ^17^, ^20^ It occurred in 15 patients (93.8%) in the FAS population, and 12 patients (92.3%) in the 300 mg/m^2^ cohort. This AE was well‐controlled using oral levothyroxine. All cases of hypothyroidism recovered promptly after the cessation of oral bexarotene.

### Dyslipidemia

Hypertriglyceridemia occurred in 13 patients (81.3%) in the FAS population and 11 patients (84.6%) in the 300 mg/m^2^ cohort. Hypercholesterolemia occurred in 13 patients (81.3%) in the FAS population and 10 patients (76.9%) in the 300 mg/m^2^ cohort. All patients with these AE recovered as a result of dose reduction or the withdrawal of oral bexarotene. In rare cases, hypertriglyceridemia may progress to acute pancreatitis. No patients developed acute pancreatitis during this study period.

### Leukopenia/neutropenia

Leukopenia and neutropenia occurred in 11 patients (68.8%) and in nine patients (56.3%) in the FAS population, respectively. Both AE occurred more frequently in the 300 mg/m^2^ cohort (Table 2). As shown in Table 3, grade 3 neutropenia can occur after treatment with bexarotene for over a year. Therefore, regular blood tests are recommended even when the treatment with bexarotene lasts for years. All patients with these AE recovered as a result of dose reduction or withdrawal of the bexarotene.

Neutropenia may occur as a DLT at all stages of bexarotene therapy. Bexarotene‐induced leukopenia may be different from bone marrow suppression caused by cytotoxic agents, because of the neutrophil proliferation and differentiation regulated by RXR‐α expression.^21^ Dose reduction of bexarotene would help bring about early improvement in the neutrophil count. Granulocyte colony‐stimulating factor treatment may be required for aged patients or patients who were treated by systemic polychemotherapy.

### Other AE

In one of the 16 patients (6.3%), serum transaminase levels were elevated to grade 3 (Tables 2,3). Only one patient with grade 2 renal dysfunction discontinued the study at week 41 of the B‐1201 trial.

In the B‐1201 trial, major treatment‐related AE such as hypothyroidism, hypercholesterolemia and hypertriglyceridemia were observed at a frequency similar to that of the B‐1101 trial. These AE occurred in an early period after the initiation of oral bexarotene. No patient discontinued this trial because of a DLT. No patients died during the study periods of the B‐1101 and B‐1201 trials.

In conclusion, the present study showed the long‐term safety of oral bexarotene for Japanese patients with CTCL. Adequate strategic management of bexarotene‐related AE will allow for the long‐term administration of bexarotene. Oral bexarotene may also induce long‐term efficacy in some subsets of Japanese CTCL patients.

## Conflict of Interest

T. H., Y. T., M. S. and K. I. have received speaker fees from Minophagen Pharmaceutical. M. S. and T. S. have received consultancy fees from Minophagen Pharmaceutical. T. H. and M. S. have received research grants and funds for research.
